# Artificial induction of third-stage dispersal juveniles of *Bursaphelenchus xylophilus* using newly established inbred lines

**DOI:** 10.1371/journal.pone.0187127

**Published:** 2017-10-26

**Authors:** Suguru E. Tanaka, Takuya Aikawa, Yuko Takeuchi-Kaneko, Kenji Fukuda, Natsumi Kanzaki

**Affiliations:** 1 Laboratory of Forest Botany, Graduate School of Agricultural and Life Sciences, the University of Tokyo, Bunkyo-ku, Tokyo, Japan; 2 Tohoku Research Center, Forestry and Forest Products Research Institute (FFPRI), Morioka, Iwate, Japan; 3 Laboratory of Terrestrial Microbial Ecology, Graduate School of Agriculture, Kyoto University, Sakyo-ku, Kyoto, Japan; 4 Kansai Research Center, FFPRI, Fushimi, Kyoto, Japan; University of Copenhagen, DENMARK

## Abstract

The pine wood nematode, *Bursaphelenchus xylophilus*, is the causal agent of pine wilt disease. This nematode has two developmental forms in its life cycle; i.e., the propagative and dispersal forms. The former is the form that builds up its population inside the host pine. The latter is specialized for transport by the vector. This form is separated into two dispersal stages (third and fourth); the third-stage dispersal juvenile (J_III_) is specialized for survival under unfavorable conditions, whereas the fourth-stage juvenile (J_IV_), which is induced by a chemical signal from the carrier *Monochamus* beetle, is transported to new host pines and invades them. Because of its importance in the disease cycle, molecular and chemical aspects of the J_IV_ have been investigated, while the mechanism of J_III_ induction has not been sufficiently investigated. In an effort to clarify the J_III_ induction process, we established inbred lines of *B*. *xylophilus* and compared their biological features. We found that the total number of nematodes (propagation proportion) was negatively correlated with the J_III_ emergence proportion, likely because nematode development was arrested at J_III_; i.e., they could not develop to adults via the reproductive stage. In addition, J_III_ induction seemed to be regulated by a small number of genes because the J_III_ induction proportion varied among inbred lines despite the high homozygosity of the parental line. We also demonstrated that J_III_ can be artificially induced by the nematode’s secreted substances. This is the first report of artificial induction of J_III_ in *B*. *xylophilus*. The *dauer* (dispersal) juvenile of the model organism *Caenorhabditis elegans* corresponds functionally to J_III_ of *B*. *xylophilus*, and this stage is known to be induced by a chemical signal referred to as daumone, derived from the nematodes’ secretion. The artificial induction of J_III_ suggests the presence of daumone-like material in *B*. *xylophilus*.

## Introduction

**Fig 1:**
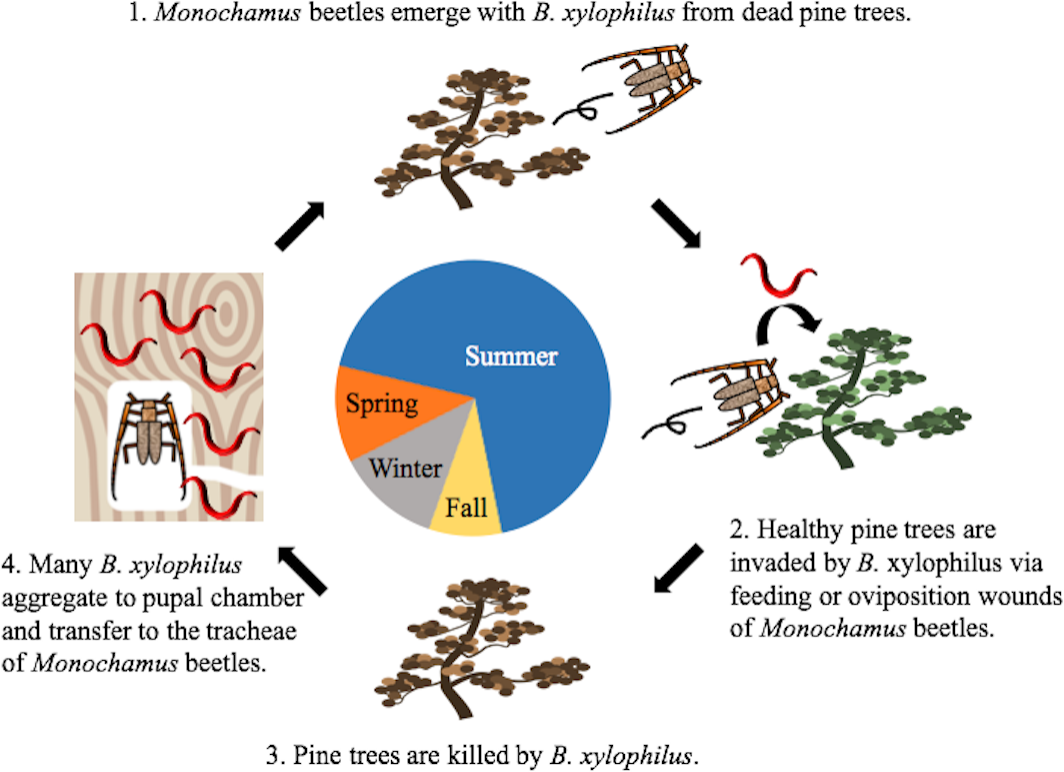
The disease cycle of pine wilt associated with pine trees, nematodes, and beetles. The center circle indicates seasons, although the length of the arc does not correspond to the actual span of the seasons in Japan.

Nematodes of the genus *Bursaphelenchus* have various types of associations with plants [1–3] and insects [4–7]. Among them, the pine wood nematode (PWN), *Bursaphelenchus xylophilus*, which causes pine wilt disease, is one of the most important forest pathogens [1, 8–13]. This mycetophagous/plant-parasitic nematode is native to North America [14–16], and is now spreading through East Asian and European countries [17]. This nematode invades host pine trees through maturation feeding or oviposition wounds caused by the vector *Monochamus* longhorn beetle [18, 19], propagates enormously, and kills the host pine [12]. *Monochamus* females oviposit on the weakened or newly dead tree, and the offspring beetle larvae develop while feeding on inner bark tissue. In the following season, adult beetles that have loaded nematodes in their tracheal system emerge from the dead pine tree, and spread the disease to healthy pine trees (Fig 1) [20, 21].

**Fig 2:**
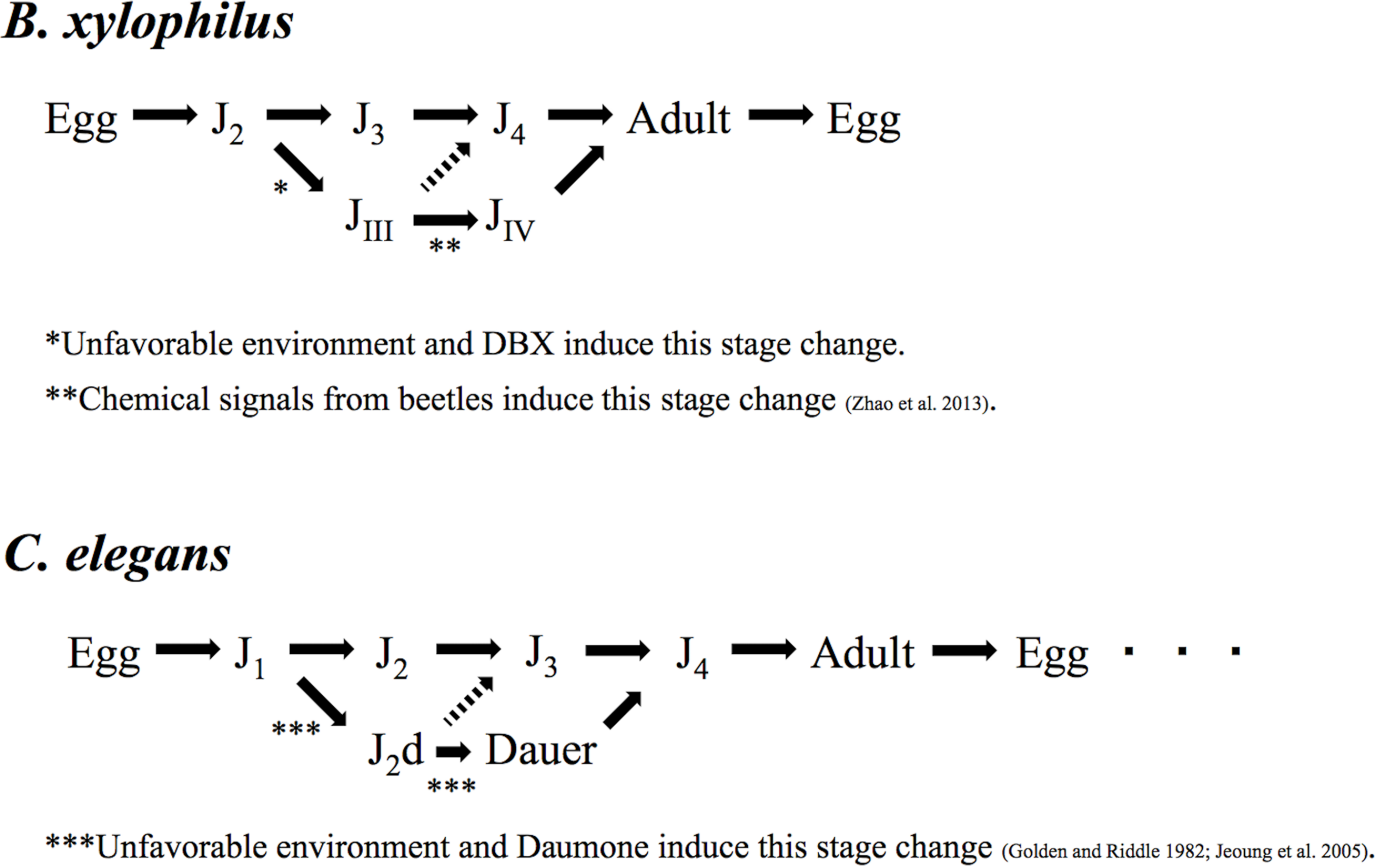
The life cycles of the pine wood nematode and *Caenorhabditis elegans*. In addition to regular ‘propagative’ (microbe-feeding) stages, each has ‘dispersal (J_III_ and J_IV_)/dormant (J_2_d and Dauer)’ stages.

The nematode *B*. *xylophilus* has a distinct life cycle; i.e., there are four propagative and two dispersal juvenile stages (Fig 2) [22]. The first-stage juvenile molts to the second stage (J_2_) in the egg, and hatches as a J_2_. Under favorable conditions, J_2_ develops into third-stage propagative juvenile (J_3_) to become an adult (male or female) via the fourth-stage propagative juvenile (J_4_). However, under unfavorable conditions, e.g., high temperature, starvation, and high population density, a J_2_ will molt to third-stage dispersal juveniles (J_III_) instead of to J_3_ [23, 24]. J_III_ can survive under unfavorable conditions such as starvation inside the dead wood of host tree from autumn to the following spring. Morphologically, J_III_ is not clearly different from J_3_, except that its body is a little larger and there is an accumulation of many lipid droplets. J_III_ molt to fourth-stage dispersal juveniles (J_IV_) when the juvenile receives a chemical signal released by the last instar larvae and pupae of the *Monochamus* beetle [23, 25, 26]. J_IV_ is morphologically equivalent to *dauer* juveniles of the model organism *Caenorhabditis elegans*, i.e., the J_IV_ has a degenerate stylet (feeding structure), poorly developed median bulb, larger cuticle layer, and sharp tail shape, and also has dome-shaped lip. These morphological characters suggest that J_IV_ is physiologically similar to the *dauer* of *C*. *elegans*, i.e., dormant stage [22]. J_IV_ moves into the tracheae of the beetle, and the nematodes are transported to new host trees to spread the disease [27]. This phoretic relationship has been investigated intensively from ecological and physiological standpoints, and the chemical and molecular biological aspects of the mechanism of J_IV_ induction have been studied because of their importance in the disease cycle [26–31]. However, compared with the J_IV_, the J_III_ stage has not been sufficiently investigated for its biological characteristics and induction, despite its importance for nematode dispersal. J_III_ is the only stage that molts to J_IV_ (i.e., J_III_ formation is a prerequisite for J_IV_ formation) and it can survive for a long period, which allows it to hide inside trees and thus become a source of the pathogen. Therefore, the mechanisms and detailed conditions for J_III_ induction must be clarified to further our understanding of vector association and transportation of PWNs.

The model organism *C*. *elegans* has a specific stage, called the *dauer* stage [32], which is morphologically and functionally similar to the dispersal stages of *B*. *xylophilus* [24, 33]. *Dauer*-stage worms can survive for about 3 months, which is much longer than the propagative life span of the worm (ca. two weeks), and this stage is induced by the chemical signals that indicate lack of food, bacteria, and high population density [34–36]. *Dauer*-inducing pheromone (daumone) is the main substance signaling nematode population density [37]. Small molecule pheromones, called ascarosides, which induce daumone, are associated with various behaviors such as gender-specific attraction, repulsion, aggregation, olfactory plasticity, and *dauer* induction [36, 38–44]. Several studies have suggested that J_III_ of *B*. *xylophilus* is induced by a mechanism similar to the *dauer* induction of *C*. *elegans*, i.e., *B*. *xylophilus* is assumed to produce daumone-like materials [45].

In this study, as a first step in the investigation of J_III_ induction, we established several inbred lines of *B*. *xylophilus* with various J_III_ formation proportions, and developed a method for artificial induction of J_III_ using crude extracts of cultured nematodes, which is expected to include a *dauer*-inducing pheromone-like substance, to understand J_III_ induction in this nematode.

## Materials and methods

### Nematode

In this experiment, four Japanese field isolates of *B*. *xylophilus*, namely, virulent S10, T4, and Ka4, and avirulent C14-5, which is physiologically and biologically different from other strains and generally used as a comparative strain for other highly virulent strains [46, 47], were used as the candidate nematode materials for the following experiments. These isolates were considered as candidates for the parent strains of inbred lines with high J_III_-emerging proportions. S10, T4, and Ka4 were originally collected from dead trees of the Japanese red pine, *Pinus densiflora*, in the Shimane Prefecture in 1982, the Iwate Prefecture in 1992, and the Ibaraki Prefecture in 1994, respectively. C14-5 was obtained from an adult *M*. *alternatus* beetle in Chiba Prefecture in 1975 [48]. These isolates have been maintained for more than 20 years as laboratory cultures in the Forestry and Forest Products Research Institute, and thus, no special permit was required to use them. For S10, T4, and Ka4, nematodes were reared on a fungal lawn of *Botrytis cinerea* on potato dextrose agar (PDA) media, and kept at 25°C for 7 days, while C14-5 was kept at 25°C for 16 days because the propagation of C14-5 is slower.

Hereafter, we define isolate as a field-collected population without genetic purification, and inbred line as the genetically purified population derived from the field population (= isolate).

### Investigation of the emerging proportion of J_III_

Propagated nematodes were collected using the Baermann funnel technique, and surface-sterilized using lactic acid following the method described in Mamiya et al. [49]. In short, nematodes were collected in a 10-mL glass tube, washed three times with distilled water, and the same volume of ca. 6% lactic acid solution was added to the nematode suspension. After 30 sec of soaking the nematodes in the lactic acid solution, the suspension was centrifuged at 1,500 rpm for 30 sec, and the supernatant was removed. Nematodes were then rinsed three times with sterilized water. Fifty surface-sterilized nematodes of each isolate were reared on the hyphae of *Cosmospora viridescens*, which is frequently used in experiments involving dispersal stage induction [50, 51], growing on malt extract agar (2% malt extract, 1.5% agarose) in 4-cm-diameter Petri dishes, and kept at 25°C for 10, 20, and 30 days. Each treatment was repeated eight times.

Then, nematodes were collected using the Baermann funnel technique, and killed by heat (ca. 60°C for 1 min), and fixed in TAF (2% triethanolamine, 2.775% formaldehyde) [52]. Fixed nematodes were kept at room temperature until microscopic observation. The numbers of total nematodes and J_III_ were counted for each sample using a light microscope (Eclipse 80i, Nikon), and the proportion of J_III_ was calculated.

### Establishment of inbred lines and evaluation of their J_III_ emerging proportion

Field-collected isolates of *B*. *xylophilus* usually have high genomic diversity [53]; therefore, it is necessary to establish inbred lines for molecular and biochemical analyses. We selected and used the isolate T4, which showed the highest J_III_ formation proportion in the above-mentioned test, as the parental isolate.

*Botrytis cinerea* was inoculated on 4% plain (water) agar in 48-well microplates, and kept at 25°C for 1 day. Distilled water (400 μL) was added on a fungal mat in the microplate, and one T4 J_III_ nematode was inoculated into the water in each well, and reared for 2 days at 25°C to obtain unmated adult females and males. *Botrytis cinerea* was cultured on 4% plain agar in 4-cm-diameter Petri dishes for 1 day. An unmated female and unmated male were then transferred to fungal hyphae on the dish. The culture plates were kept at room temperature and observed every day, and a young gravid female of the next generation was transferred to a new dish. Offspring were isolated using a microplate and used for the next mating. This procedure was repeated for 12 generations, and eight inbred lines were thus established from T4, and coded as ST1 to ST8.

The J_III_ formation proportions of the eight newly established inbred lines were examined with the method described above.

### Extraction of J_III_-inducing pheromone

Based on studies of *C*. *elegans* and *B*. *xylophilus*, we hypothesized that *B*. *xylophilus* has a substance like daumone, which is a heat-tolerant and water-soluble material [18, 35, 37, 45], and tentatively named it DBX for daumone of *B*. *xylophilus*. Therefore, we prepared crude DBX for artificial induction of J_III_. In this experiment, we used the ST2 line, which had the highest J_III_ production proportion in the J_III_ induction test. ST2 nematodes were reared for four days on *B*. *cinerea* grown on 10-mL barley grains in a 50-mL Erlenmeyer flask. Nematodes were collected using the Baermann funnel technique for three hours, rinsed with distilled water three times, and a sterilized water suspension of the nematodes (10 worms/μL) was prepared. The suspension was shaken using a Triple Shaker NR-80 (Taitec, Japan) at 95 rpm for 48 hours at 25°C, and the suspension was filtrated using filter paper (Kiriyama Filter paper No. 5A, 7 μm; Kiriyama Glass, Tokyo) twice to remove worms. The filtered solution supposedly containing DBX was concentrated by freeze-drying, and the dried materials were dissolved in sterilized water, adjusting to 2,000,000 nematodes’ secretions per 3 mL. The solution was tentatively called CDBX for crude DBX.

### Artificial induction of J_III_

CDBX (200 μL) was inoculated on the surface of 4% plain agar in 4-cm Petri dishes, and 20 μL of yeast (*Saccharomyces cerevisiae*; 0.25 mg/mL or 1 mg/mL) suspension was added as food for the nematodes. In the control group, the same volume of sterilized distilled water was added instead of CDBX, along with the same amount of yeast. The plates were kept for two days at 25°C. Thereafter, ten females and three males of ST2 were reared on each plate, and cultured for 5 days. Each treatment was repeated three times. After confirming the propagation (presence of offspring), we extracted the nematodes using the Baermann funnel technique overnight and counted the number of total and J_III_ nematodes to calculate the J_III_ proportion.

In addition to the above experiment, we examined J_III_ formation under different conditions: without higher food concentrations (4 mg/mL suspension of *S*. *cerevisiae*), and with a shorter incubation (4 days) for all treatments. After four or five days of incubation, all juvenile nematodes were first generation progenies because the life cycle of *B*. *xylophilus* on yeast is longer than five days at 25°C [22].

### Statistical analysis

The statistical analysis was performed using R ver. 3.2.4. We calculated the emergence proportion of J_III_ using the following formula:

$$Proportion\;\left(\%\right)=\left[\left(number\;of\;J_{III}\right)\;/\;\left(total\;number\;of\;worms\right)\right]\;\times \;100$$

Data represent the mean ± standard error (SE). For the statistical analysis, we performed the normality test to reveal the normality of the data to decide whether to use parametric or non-parametric tests. For the J_III_ emergence proportion of the isolates, Kruskal–Wallis one-way analysis was performed to test among isolates, and subsequent Steel–Dwass multiple comparison tests were conducted. To analyze J_III_ emergence proportions of inbred lines, one-way analysis of variance (ANOVA) and post hoc Tukey–Kramer multiple comparison tests were conducted. When analyzing artificial induction of J_III_ tests, one-way ANOVA and post hoc Tukey–Kramer multiple comparison tests were performed. To analyze the correlation of all numbers and J_III_ proportions, we conducted Spearman’s rank correlation test.

## Results

### Proportion of J_III_ formation in the conventional isolates

**Fig 3:**
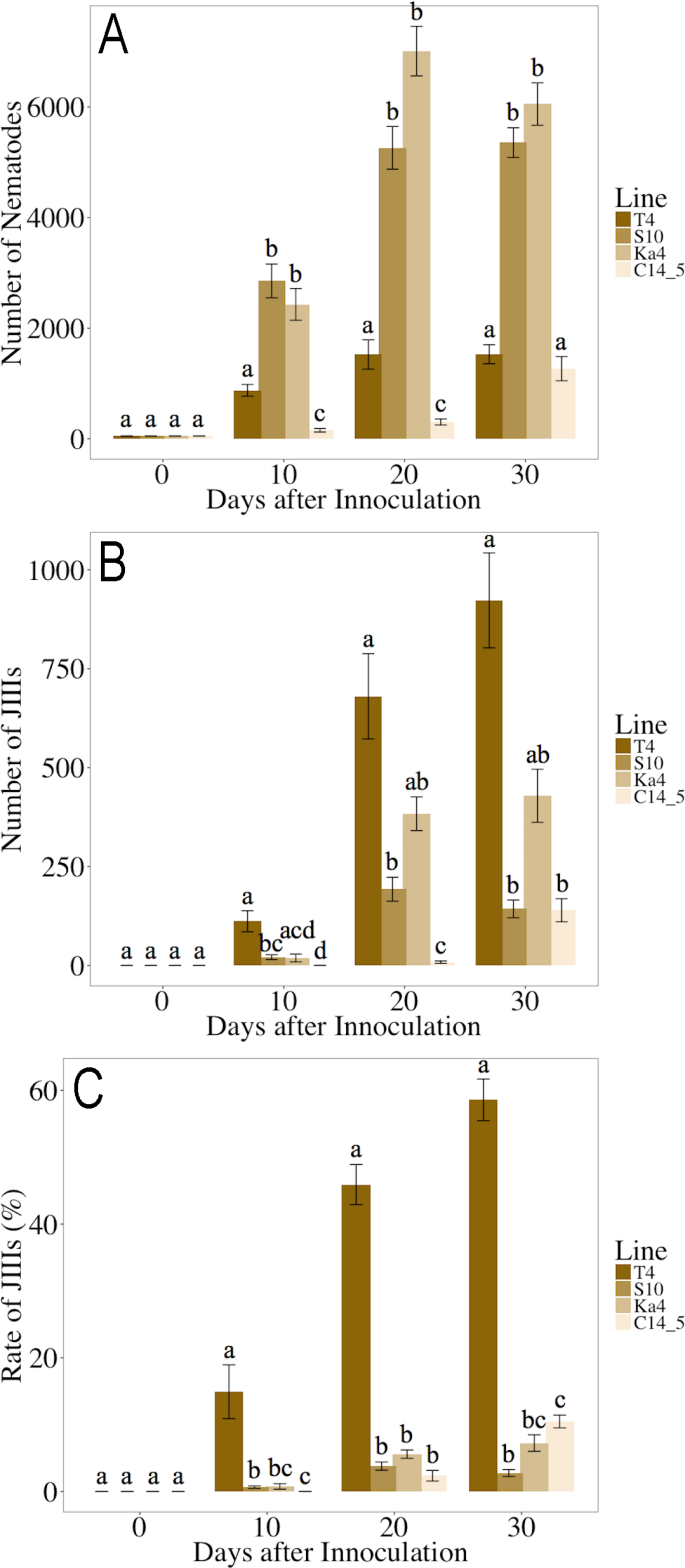
Propagation and J_III_ production of four field isolates of *Bursaphelenchus xylophilus* after 10, 20, and 30 days of incubation. A: total number of nematodes; B: number of J_III_; C: J_III_ proportion. The same letters indicate non-significant differences between the samples at the same time point. Bars and error bars represent averages and standard errors for ten replicates, respectively.

The results are summarized in Fig 3 and Supplementary S1 Table. Two virulent isolates, S10 and Ka4, propagated rapidly, and maintained high population densities over the 30 days of the experimental period. Conversely, virulent T4 and avirulent C14-5 propagated slowly (*P* < 0.01). However, the numbers and proportions of J_III_ were significantly higher in T4 compared with the other three isolates (*P* < 0.01 for both).

### Proportion of J_III_ formation in inbred lines

**Fig 4:**
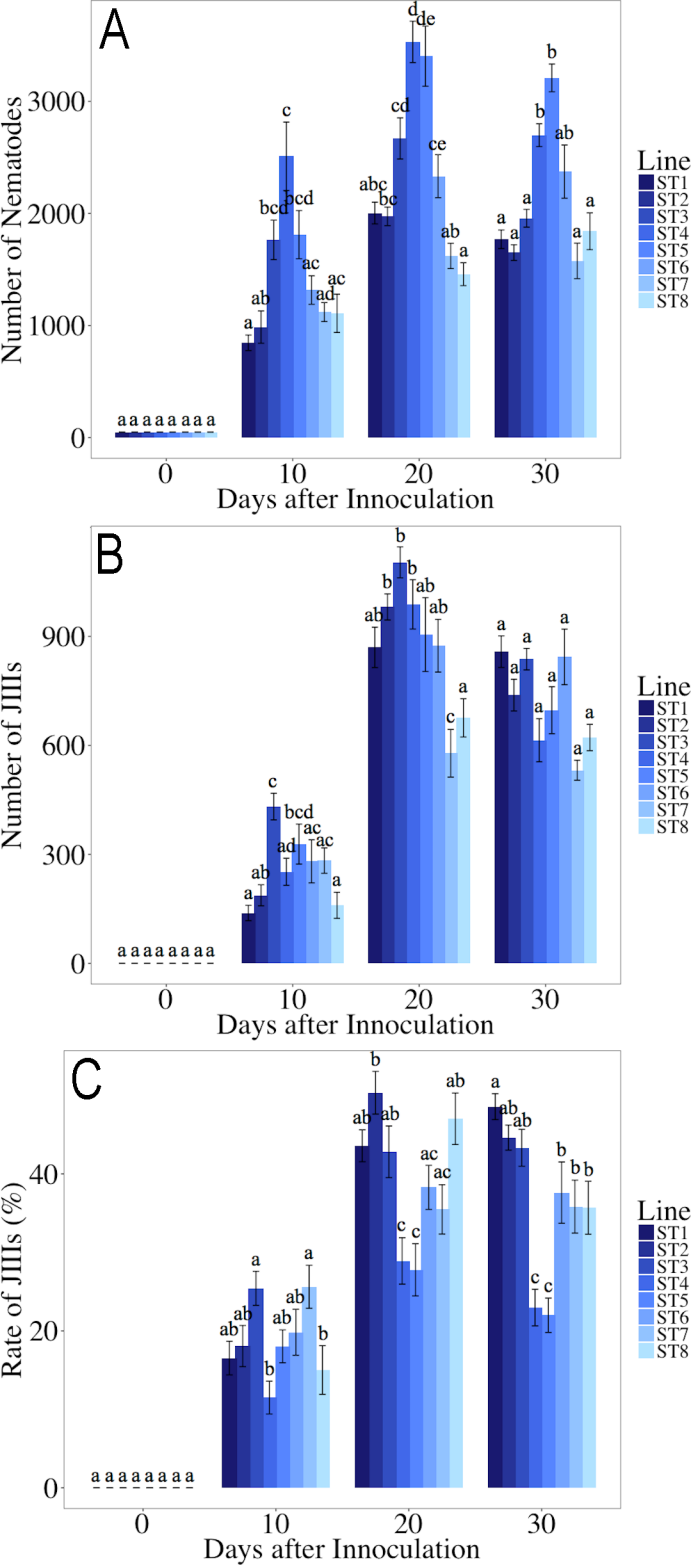
Propagation and J_III_ production of eight inbred lines of *Bursaphelenchus xylophilus* derived from the T4 isolate after 10, 20, and 30 days of incubation. A: total number of nematodes; B: number of J_III_; C: J_III_ proportion; D: correlation between number of total nematodes and J_III_ proportion. The same letters indicate non-significant differences between the samples at the same time point (A–C). Bars and error bars represent averages and standard errors for ten replicates, respectively (A–C).

**Fig 5:**
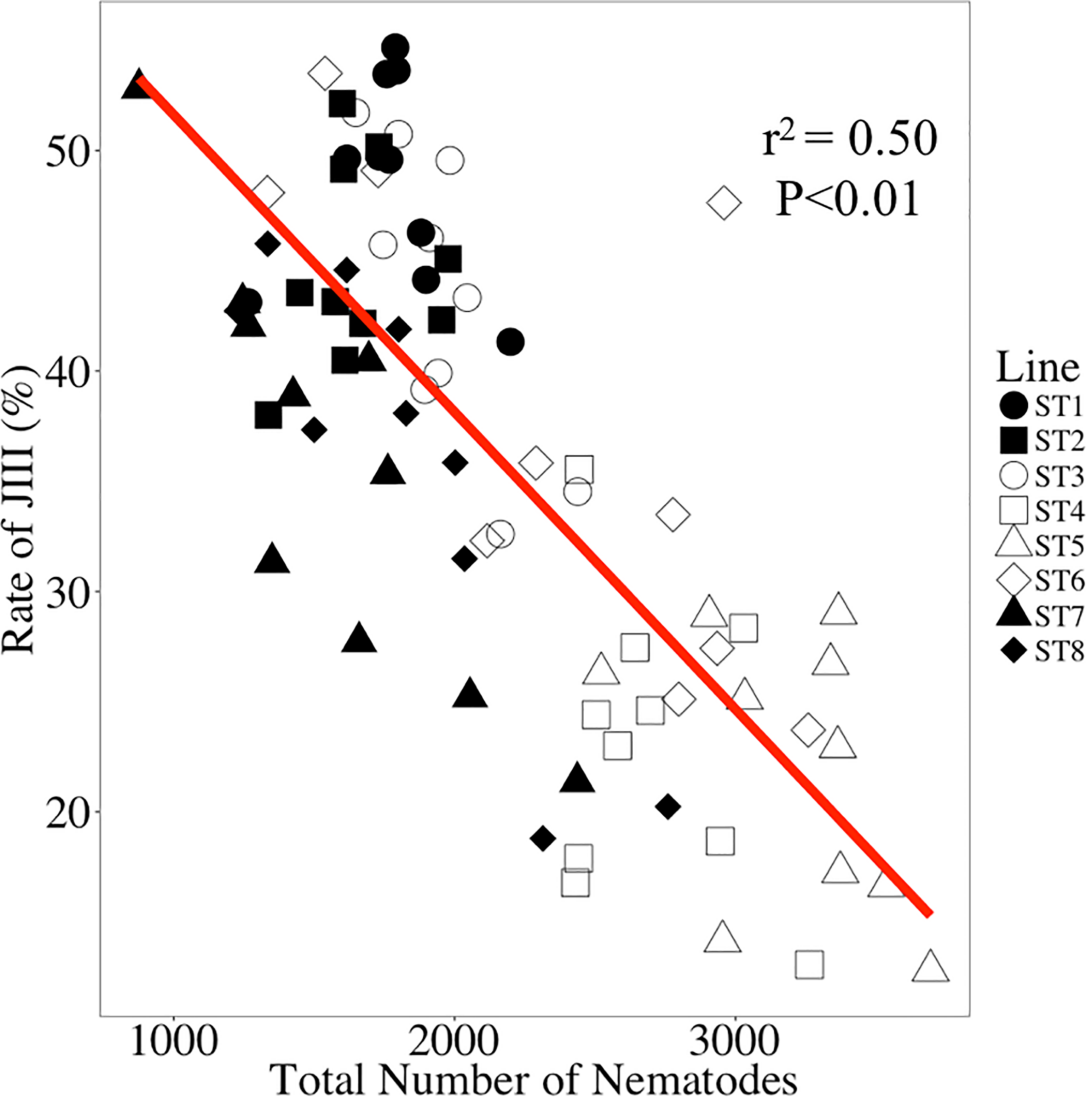
Correlation between number of total nematodes and J_III_ proportion. Each plot represents a culture plate.

The results are summarized in Fig 4, and Supplementary S2 Table. The total number of nematodes in lines ST4 and ST5 was significantly higher than in the other six lines, while the J_III_ formation proportion of ST1 and ST2 was significantly higher than in the other lines, and that of ST4 and ST5 was significantly lower than the others. This tendency was supported by the significant negative correlation between the total number of nematodes and J_III_ formation proportion (*r*^*2*^ = 0.50, *P* < 0.01; Fig 5).

### Artificial induction of J_III_

**Fig 6:**
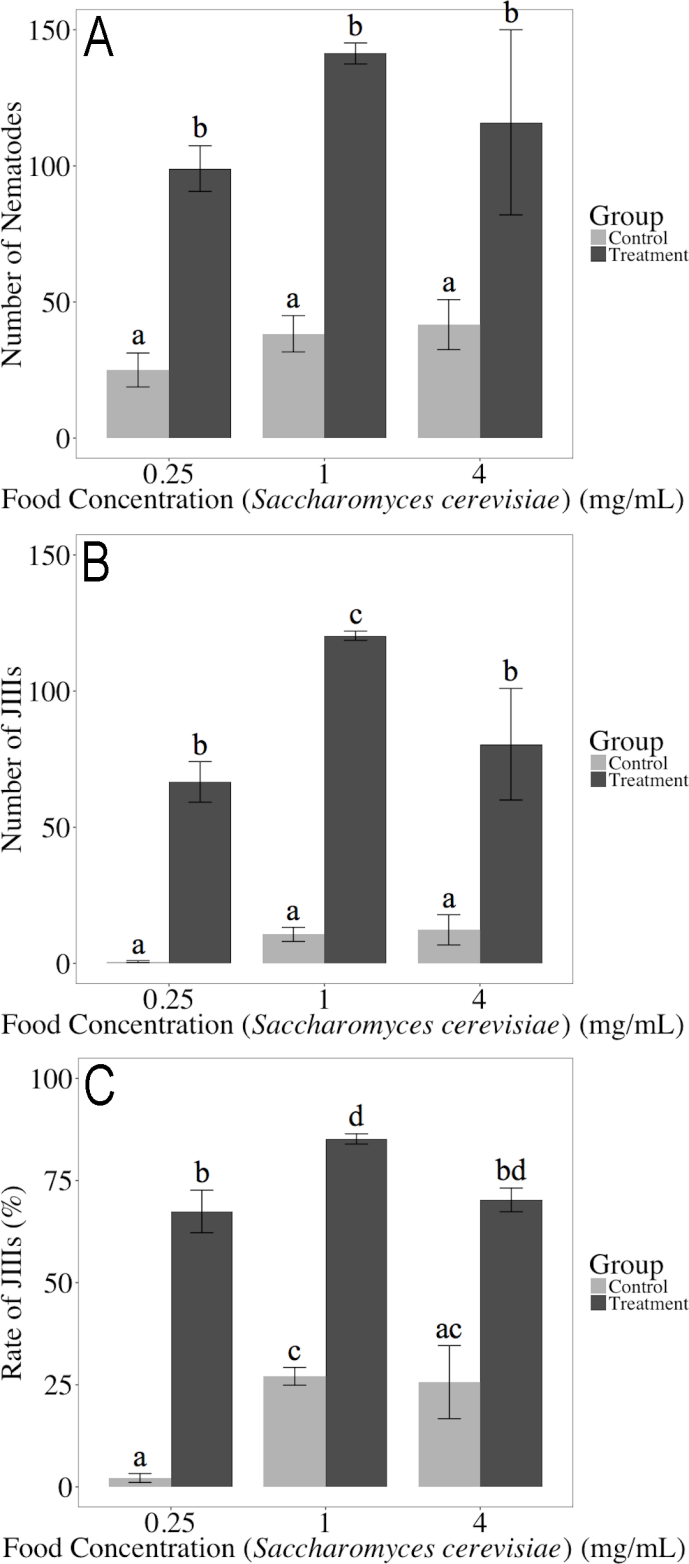
Propagation and J_III_ production of the ST2 line of *Bursaphelenchus xylophilus* by adding CDBX and various concentrations of yeast (*Saccharomyces cerevisiae*) after five days of incubation. A: total number of nematodes; B: number of J_III_; C: J_III_ proportion. The same letters indicate non-significant differences between the samples. Bars and error bars represent averages and standard errors for three replicates, respectively.

The results are summarized in Fig 6 and Supplementary S4 Table. The total number of nematodes was higher in the treatment group than the control group (*P* = 0.00001809), and the proportion of J_III_ was also higher in the treatment group than in the control group (*P* = 0.0000002041). We also checked the 4-day incubation experiment. The results showed a similar tendency, i.e., the total number of nematode and the proportion of J_III_ were higher in the treatment group than the control group (S1 Fig and S3 Table). When observing the plates, the nematodes that remained were mostly J_2_ (data not shown).

## Discussion

Many isolates of *B*. *xylophilus* have been isolated and maintained in laboratories, and are quite variable in some biological characteristics, *e*.*g*., virulence, propagation, and efficiency of phoresy [46]. However, regardless of its importance, J_III_ formation proportion has not been closely compared among isolates. In this study, a T4 isolate showed a significantly higher J_III_ formation proportion than the other three isolates (Fig 3, S1 Table). Interestingly, although the T4 isolate is known to propagate quickly [54], its population size was significantly smaller than the other two pathogenic isolates throughout the experiment. In previous studies, J_III_ began to appear after the nematode population reached the maximum level, and the proportion and number of J_III_ increased as the population decreased [23, 24, 55]. Considering the relationship between population size and J_III_ formation proportion, nematodes of T4 are suspected to be more sensitive to J_III_ induction signals, such as unfavorable conditions and the concentration of DBX, than other isolates, and they shifted to the dispersal life cycle in the early phase of incubation. In comparison, most individuals of the S10 and Ka4 isolates were J_2_ after 30 days of the experimental period (S5 Table). This suggests that the nematodes of these two isolates, S10 and Ka4, were starved and suspended their growth before turning to J_III_. In the model organism nematode *C*. *elegans* four distinct pathways that regulate *dauer* arrest have been confirmed by analyses of *dauer*-constitutive and *dauer*-defective mutants (guanylyl cyclase pathway, TGFβ-like pathway, insulin-like pathway, and steroid hormone pathway) [56–62]. These analyses demonstrated that the ability to form *dauer* differs among mutants. Further, a satellite model species, *Pristionchus pacificus*, has natural variation in pheromone production and sensitivity derived from genetic differentiation among several strains collected from different localities all over the world [63, 64]. The variation of J_III_ formation among PWN isolates seems similar to that of *dauer* formation among *C*. *elegans* and *P*. *pacificus* strains, and therefore, nematodes of the isolate T4 may have some mutations associated with J_III_ formation so they more readily form J_III_ compared with other isolates. In this study, we could not reveal which production or sensitivity of pheromone resulted in the high J_III_ proportion in T4; further physiological and chemical analyses are required to reveal the answer. It is interesting that T4 nematodes have maintained a high J_III_ production ability for a long sub-culturing history, despite extended sub-culturing of PWN isolates sometimes causing reductions in the pathogenicity against host pine trees and decreases in the proportion of J_III_ formation [24, 65].

In a previous study, T4 showed a high degree of homozygosity (only 4.26% of variants in the T4 isolate genome) [66], suggesting that the genetic diversity is rather low in this isolate. However, in the present study the proportion of J_III_ formation varied among inbred lines derived from the isolate; i.e., T4 retains genetic diversity associated with J_III_ formation within the highly homozygous isolate. The number of *dauer*-related (*daf*) genes varies among the nematode groups. For example, *C*. *elegans* has 1,259 *daf* genes in the latest version of gene association analysis (WS258) (http://www.wormbase.org), and only 52 *daf*-gene groups were identified in *Pristionchus pacificus* (http://www.wormbase.org). In the case of T4, although the isolate has low genetic diversity, the J_III_-related genes seem variable among inbred lines because of their discontinuous J_III_ formation proportions, i.e., the J_III_ emerging proportions of inbred lines were separated statistically into high (ST1, ST2, and ST3), medium (ST6, ST7, and ST8), and low (ST4 and ST5) proportion groups (Fig 4). Therefore, J_III_ formation of T4 is likely regulated by a relatively small number of key genes as in *P*. *pacificus*.

The total number of nematodes was negatively correlated with the J_III_ proportion; i.e., the lines showing a high J_III_ formation proportion did not propagate well during the experimental period. This negative correlation is probably because of the developmental arrest in J_III_. J_III_ is a kind of *dauer* (dormant) stage, and thus the individual that molted to J_III_ does not develop to the reproductive stage (= adult) under certain conditions. Therefore, the next generation of nematodes was produced only by the adults that had emerged at an earlier stage of culture (= early generation of adults). However, in the lines in which the J_III_ formation proportion was lower, nematodes developed to adults and reproduced more effectively than the lines with higher J_III_ formation, and the total nematode population became larger in such lines.

After the first-generation adults were exposed to CDBX, most offspring of the next generation (J_2_) molted to J_III_, suggesting the presence of water-soluble material(s) that induce J_III_ formation. In *C*. *elegans*, daumone was first identified as ascaroside (–)- 6-(3,5-dihydroxy-6-methyltetrahydropyran-2-yloxy) heptanoic acid [37]; i.e., an ascarylose component with a fatty acid side chain that can be dissolved in water. Ascarosides are often used as signaling molecules, such as *dauer*-inducing pheromone, in various nematode species including free-living and parasitic species (*C*. *elegans*, *C*. *afra*, *Heterorhabditis bacteriophora*, *Allodiplogaster seani*, *Nippostrongylus brasiliensis*, *Oscheius carolinensis*, *O*. *tipulae*, *Parastrongyloides trichosuri*, *Pelodera strongyloides*, *P*. *pacificus*, *Rhabditis* sp. AF5, *Steinernema carpocapsae*, *S*. *glaseri*, and *S*. *riobrave*) [67–71]. Therefore, CDBX as a J_III_ inducer can be hypothesized to include ascaroside-like substances, which can induce the J_III_ formation of *B*. *xylophilus*. It was reported that the *dauer*-inducing pheromones of *C*. *elegans* and *P*. *pacificus* did not induce *dauer* formation of *P*. *pacificus* and *C*. *elegans*, respectively, although the physical properties of those two pheromones were similar to one another [72]. This suggests that *dauer*-inducing pheromones are species-specific or affect only closely related (narrow-ranged) species; that of *B*. *xylophilus* should be no different. Further physiological analyses on the J_III_ induction of *B*. *xylophilus* will be necessary to reveal this species specificity.

## Conclusion

*Bursaphelenchus xylophilus*, the causal agent of pine wilt, is an important forest pathogen. However, the mechanism of induction of dispersal stages has not been studied in detail despite its importance for expansion of the disease. In the present study, we clarified that 1) the manner of J_III_ formation is different among field isolates of *B*. *xylophilus*, 2) a set of newly established inbred lines varied in J_III_ production proportion, and 3) water soluble substances of nematode secretion induced J_III_ production. The inbred lines derived from the T4 isolate may be useful for investigating J_III_ formation in future studies, e.g., RNA-sequencing analyses of nematodes after J_III_ induction treatment to identify the genetic pathways of J_III_ formation. Furthermore, the J_III_ formation assay system established here will be applicable to chemical analyses of DBX.

J_III_ of *B*. *xylophilus* (and its close relatives) is an interesting stage because it is induced in a similar manner to *dauer*s of other nematode species, and has biological characteristics similar to *dauer*s, e.g., long-term survival [45, 73, 74]. However, in *B*. *xylophilus*, it is not J_III_, but rather J_IV_, that is the true *dauer* stage [18, 75]. By conducting further analyses of the molecular biology and chemistry associated with J_III_ formation, information that is important not only for disease control, but also for developmental biology, will be revealed.

## Supporting information

S1 Fig Propagation and J_III_ production of the ST2 line of *Bursaphelenchus xylophilus* by adding CDBX and various concentrations of yeast (*Saccharomyces cerevisiae*) after 4 days of incubation. A: total number of nematodes; B: number of J_III_; C: J_III_ proportion. The same letters indicate non-significant differences between the samples. Bars and error bars represent averages and standard errors for three replicates, respectively.
(TIF)

S1 Table The number of total and J_III_ nematodes, and the J_III_ emerging proportion of four field isolates after 10, 20, and 30 days of incubation. Values are average ± standard error (SE) of ten replicates.
(DOCX)

S2 Table The number of total and J_III_ nematodes and the J_III_ emerging proportion of eight inbred lines after 10, 20, and 30 days of incubation. Values are average ± SE of ten replicates.
(DOCX)

S3 Table Propagation and J_III_ production of the ST2 line of *Bursaphelenchus xylophilus* by adding CDBX and various concentrations of the yeast *Saccharomyces cerevisiae* after 4 days of incubation. Values are average ± SE of three replicates.
(DOCX)

S4 Table Propagation and J_III_ induction of ST2 line of *B*. *xylophilus* by adding CDBX and various concentrations of the yeast *S*. *cerevisiae* after five days of incubation. Values are average ± SE of three replicates.
(DOCX)

S5 Table The number of each stage of two field isolates (S10 and Ka4) after 30 days of incubation. Values are average ± SE of three replicates.
(DOCX)
