# Supplementary figures and images for: Artificial induction of third-stage dispersal juveniles of Bursaphelenchus xylophilus using newly established inbred lines
Source: PLoS One. 2017 Oct 26;12(10):e0187127. doi: 10.1371/journal.pone.0187127 (PMC5658132; doi:10.1371/journal.pone.0187127)

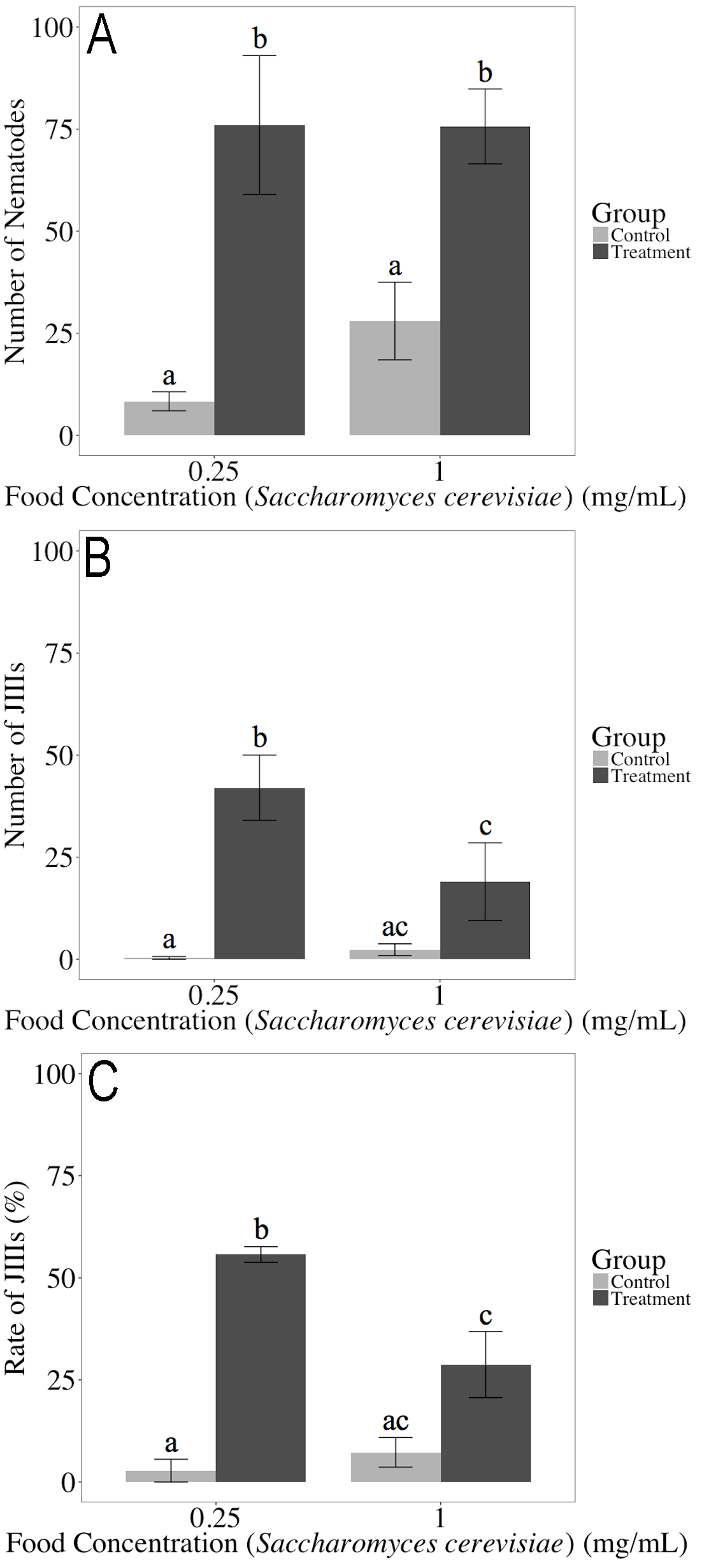

Supplement: S1 Fig — A: total number of nematodes; B: number of JIII; C: JIII proportion. The same letters indicate non-significant differences between the samples. Bars and error bars represent averages and standard errors for three replicates, respectively. (TIF) [file pone.0187127.s001.tif]
