# Supplementary material for: Artificial induction of third-stage dispersal juveniles of Bursaphelenchus xylophilus using newly established inbred lines
Source: PLoS One. 2017 Oct 26;12(10):e0187127. doi: 10.1371/journal.pone.0187127 (PMC5658132; doi:10.1371/journal.pone.0187127)
Supplement: S1 Table — Values are average ± standard error (SE) of ten replicates. (DOCX) [file pone.0187127.s002.docx]

**S1 Table. The number of total and JIII nematodes and JIII emerging rate of four field isolates after 10, 20 and 30 days of incubation.**

| **Isolate** | **Days** | **Number of all nematodes** | **Number of JIIIs** | **JIII Rate (%)** |
| --- | --- | --- | --- | --- |
| **T4** | 0 | 50.0±0.0 | 0.0±0.0 | 0.0±0.0 |
|  | 10 | 877.8±105.9 | 112.0±26.6 | 14.9±4.0 |
|  | 20 | 1524.2±265.0 | 680.2±107.9 | 45.9±3.0 |
|  | 30 | 1530.2±171.6 | 922.8±120.0 | 58.59±3.1 |
| **S10** | 0 | 50.0±0.0 | 0.0±0.0 | 0.0±0.0 |
|  | 10 | 2855.0±305.4 | 21.1±6.0 | 0.7±0.2 |
|  | 20 | 5260.8±385.8 | 192.9±30.2 | 3.8±0.6 |
|  | 30 | 5356.1±268.4 | 143.1±22.4 | 2.8±0.5 |
| **Ka4** | 0 | 50.0±0.0 | 0.0±0.0 | 0.0±0.0 |
|  | 10 | 2430.5±287.8 | 19.0±10.0 | 0.8±0.4 |
|  | 20 | 7010.7±448.7 | 383.6±42.5 | 5.6±0.6 |
|  | 30 | 6053.0±382.6 | 429.0±67.2 | 7.3±1.2 |
| **C14-5** | 0 | 50.0±0.0 | 0.0±0.0 | 0.0±0.0 |
|  | 10 | 154.5±31.8 | 0.0±0.0 | 0.0±0.0 |
|  | 20 | 304.6±55.9 | 8.46±3.2 | 2.4±0.8 |
|  | 30 | 1268.6±218.4 | 139.5±29.3 | 10.5±0.9 |

Values are in a form: average ± SE of 10 replicates.
