# Supplementary material for: Artificial induction of third-stage dispersal juveniles of Bursaphelenchus xylophilus using newly established inbred lines
Source: PLoS One. 2017 Oct 26;12(10):e0187127. doi: 10.1371/journal.pone.0187127 (PMC5658132; doi:10.1371/journal.pone.0187127)
Supplement: S2 Table — Values are average ± SE of ten replicates. (DOCX) [file pone.0187127.s003.docx]

**S2 Table. The number of total and JIII nematodes and JIII emerging rate of eight inbred lines after 10, 20, and 30 days incubation.**

| **Line** | **Days** | **Number** | **JIII Number** | **JIII Rate (%)** |
| --- | --- | --- | --- | --- |
| **ST1** | 0 | 50.0±0.0 | 0.0±0.0 | 0.0±0.0 |
|  | 10 | 846.5±69.3 | 138.6±21.0 | 16.5±2.1 |
|  | 20 | 2003.0±96.9 | 869.8±55.6 | 43.6±2.0 |
|  | 30 | 1769.7±82.8 | 858.1±43.5 | 48.6±1.7 |
| **ST2** | 0 | 50.0±0.0 | 0.0±0.0 | 0.0±0.0 |
|  | 10 | 986.7±144.1 | 187.4±28.9 | 18.1±2.6 |
|  | 20 | 1973.9±82.9 | 981.1±36.0 | 50.3±2.7 |
|  | 30 | 1650.8±70.3 | 738.4±43.5 | 44.6±1.6 |
| **ST3** | 0 | 50.0±0.0 | 0.0±0.0 | 0.0±0.0 |
|  | 10 | 1764.0±175.7 | 431.6±36.5 | 25.4±2.2 |
|  | 20 | 2668.8±183.4 | 1104.3±42.6 | 42.8±3.3 |
|  | 30 | 1956.8±79.4 | 837.5±29.7 | 43.3±2.4 |
| **ST4** | 0 | 50.0±0.0 | 0.0±0.0 | 0.0±0.0 |
|  | 10 | 2509.3±304.9 | 252.2±37.3 | 11.5±2.1 |
|  | 20 | 3530.0±184.7 | 988.1±67.8 | 28.9±3.0 |
|  | 30 | 2697.6±101.9 | 614.1±59.3 | 23.0±2.3 |
| **ST5** | 0 | 50.0±0.0 | 0.0±0.0 | 0.0±0.0 |
|  | 10 | 1810.7±214.2 | 328.4±55.1 | 18.0±2.1 |
|  | 20 | 3401.6±268.3 | 905.0±101.8 | 27.8±3.3 |
|  | 30 | 3209.0±123.4 | 696.8±64.6 | 22.0±2.2 |
| **ST6** | 0 | 50.0±0.0 | 0.0±0.0 | 0.0±0.0 |
|  | 10 | 1316.7±126.8 | 280.9±59.3 | 19.8±2.9 |
|  | 20 | 2331.6±191.5 | 874.2±72.4 | 38.3±2.8 |
|  | 30 | 2373.4±237.3 | 843.6±76.5 | 37.6±3.9 |
| **ST7** | 0 | 50.0±0.0 | 0.0±0.0 | 0.0±0.0 |
|  | 10 | 1120.2±84.8 | 283.0±35.0 | 25.6±2.7 |
|  | 20 | 1620.7±113.2 | 578.3±65.6 | 35.5±3.1 |
|  | 30 | 1577.0±157.9 | 531.6±27.5 | 35.8±3.4 |
| **ST8** | 0 | 50.0±0.0 | 0.0±0.0 | 0.0±0.0 |
|  | 10 | 1109.2±171.0 | 159.9±35.9 | 15.0±3.1 |
|  | 20 | 1457.5±102.3 | 675.7±52.7 | 47.0±3.3 |
|  | 30 | 1841.5±163.9 | 621.7±36.3 | 35.7±3.4 |

Values are in a form: average ± SE of 10 replicates.
