# Supplementary material for: Artificial induction of third-stage dispersal juveniles of Bursaphelenchus xylophilus using newly established inbred lines
Source: PLoS One. 2017 Oct 26;12(10):e0187127. doi: 10.1371/journal.pone.0187127 (PMC5658132; doi:10.1371/journal.pone.0187127)
Supplement: S3 Table — Values are average ± SE of three replicates. (DOCX) [file pone.0187127.s004.docx]

**S3 Table. Propagation and JIII production of ST2 line of *Bursaphelenchus xylophilus* by adding CDBX and various concentrations of food yeast (*Saccharomyces cerevisiae*) after 4 days of incubation.**

|  | **Food concentration (mg/mL)** | **Number of all nematodes** | **Number of JIIIs** | **JIII Rate (%)** |
| --- | --- | --- | --- | --- |
| **Treatment** | 0.25 | 76.0±17.0 | 42.0±8.0 | 55.7±0.0 |
|  | 1 | 75.7±9.2 | 19.0±9.5 | 2.8±0.0 |
| **Control** | 0.25 | 8.3±2.3 | 0.3±0.3 | 28.8±0.1 |
|  | 1 | 28±9.5 | 2.3±1.5 | 7.2±0.0 |

Values are in a form: average ± SE of three replicates.
