# Supplementary material for: Artificial induction of third-stage dispersal juveniles of Bursaphelenchus xylophilus using newly established inbred lines
Source: PLoS One. 2017 Oct 26;12(10):e0187127. doi: 10.1371/journal.pone.0187127 (PMC5658132; doi:10.1371/journal.pone.0187127)
Supplement: S4 Table — Values are average ± SE of three replicates. (DOCX) [file pone.0187127.s005.docx]

**S4 Table. Propagation and JIII induction of ST2 line of *Bursaphelenchus xylophilus* by adding CDBX and various concentrations of food yeast (Saccharomyces cerevisiae), after 5 days of incubation.**

|  | **Food concentration (mg/mL)** | **Number of all nematodes** | **Number of JIIIs** | **JIII Rate (%)** |
| --- | --- | --- | --- | --- |
| **Treatment** | 0.25 | 99±8.4 | 66.7±7.4 | 67.4±0.1 |
|  | 1 | 141.3±3.8 | 120.3±1.7 | 85.2±0.0 |
|  | 4 | 116.0±34.0 | 80.5±20.5 | 70.3±0.0 |
| **Control** | 0.25 | 25.0±6.2 | 0.7±0.3 | 2.2±0.0 |
|  | 1 | 38.3±6.7 | 10.7±2.6 | 27.1±0.0 |
|  | 4 | 41.7±9.2 | 12.3±5.5 | 25.6±0.1 |

Values are in a form: average ± SE of three replicates.
