# Supplementary material for: Artificial induction of third-stage dispersal juveniles of Bursaphelenchus xylophilus using newly established inbred lines
Source: PLoS One. 2017 Oct 26;12(10):e0187127. doi: 10.1371/journal.pone.0187127 (PMC5658132; doi:10.1371/journal.pone.0187127)
Supplement: S5 Table — Values are average ± SE of three replicates. (DOCX) [file pone.0187127.s006.docx]

**S5 Table. The number of each stage of two field isolates (S10 and Ka4) after 30days of incubation.**

| **Isolate** | **Number of nematodes** | | | | |
| --- | --- | --- | --- | --- | --- |
|  | **L2** | **L3** | **L4** | **Adult** | **JIII** |
| **S10** | 110.5±14.5 | 23.0±3.0 | 17.5±2.5 | 13.0±2.0 | 6.5±1.5 |
| **Ka4** | 101.0±1.0 | 13.0±4.0 | 17.0±2.0 | 8.5±4.5 | 5.5±0.5 |

Values are in a form: average ± SE of three replicates.
